# Whisky, as a Prophylatic in Typhoid Fever

**Published:** 1861-08

**Authors:** Hugh Kelly

**Affiliations:** Iredell County, N. C.


*Whisky as a Prophylatic in Typhoid Fever.* By Hugh Kelly, M.D., of Iredell county, N. C.

The above announcement may excite surprise, incredulity or disgust in some; but all should, however, suspend their judgement until they have had an opportunity to test it fully. I by no means advocate the regular, or even the occasional use of ardent spirits as a beverage by persons in health, for I hold that spirituous liquors are in their proper sphere, only when used and prescribed as medicines. Consequently, persons in health should not indulge, for by so doing, they tax their systems beyond what they have a right to do. Very frequently the stomach, lungs and kidneys are overworked to dispose of what is not at all congenial to health, and particularly to the neutral functions of those organs. What a very short period elapses betwixt the reception into the stomach of a portion of spirits, and efforts for relief through the lungs and kidneys, rendering it very evident that it is an intruder, all the energies of the system being called upon to dispose of this unwelcome stranger.

In the spring and summer of 1847, there was a great deal of typhoid fever in the neighborhood of Gold Hill (then a village containing a population of some eight or nine hundred inhabitants, white and black.) The fever made its appearance in the latter part of April, was of a very grave type, and was quite fatal among the blacks. The appointment of a nurse was almost equal to passing sentence of death on the one so appointed. Some four or five of them died. The whites suffered very much also, and a number of fatal cases occurred. In one instance, where some sixty or more negroes were employed by Barnhart, Manney & Co., the number of sick was between thirty-five and forty; and, in fact, all seemed to be dispirited and languid. Nurse after nurse dying, I feared that all might in a short time be confined to bed, whites as well as blacks. A short time previous to this, I had read the history of typhoid fever in an Irish Hospital, that had continued for some time, proving very fatal to nurses and physicians, and making it difficult to get those who were willing to take the places made vacant by disease. Only two or three nurses of the original corps were left, and not one physician. One of the newly appointed doctors inquired of those nurses how they managed to keep clear of the fever, their answer was that they took their whisky three times a day, and plenty of it; hence they did not fear the fever at all. The physician acting upon this hint, had all the nurses and doctors belonging to the house to commence the use of whisky, and he reported that from that time no new case occurred in the house. Finding myself to be similarly situated, I ultimately prevailed upon the company (all being temperance men) to try whisky, by giving from half a gill to a gill three times a day for an adult, and to children in proportion. I was much gratified at the happy change effected. From the day they began to give the hands, both white and black, their allowance of whisky, not a single new case occurred, and the fever subsided in some two or three weeks, and all were up again.

I was so much pleased with this result, that I determined to try it further, and did adopt it in all the families to which I was called, where they had fever of this type, from this time, July '47, up to the time of my removal, December '54. In not one single instance did a new case occur, during that period, after the family (I mean those that were able to be up,) had commenced the use of this remedy. Since my residence in Iredell, I have pursued this same course, and with the same success. Some persons in this section of country would not make a trial of this preventative, and all or nearly all of their families were sufferers. A trial was made in this neighborhood last summer, which resulted as all the rest had done. Col. A. has a large number of negroes who had the fever among them in June last. It was of a malignant type, and after some three or four deaths, he procured a barrel of whiskey and commenced giving it as heretofore directed. The result was that not another case occurred on his farm. Its *modus operandi* in preventing this fever, I leave for others to examine; that it is a preventive, I am fully satisfied from an experience of thirteen years. When called to attend on cases of this fever, I advise all the family that are on foot to use this preventive, and to continue its use for some time, at least, until the sick are out and able to take exercise; or, for two weeks, at least, after the fever subsides in the family. In many instances where several members of the same family were attacked at or about the same time, I have seen the remaining members of the household adopt this prophylatic, and continue to watch and nurse for several weeks in succession without one of them taking the fever, which fully sustains the opinion formed of this agent. I suggest to all who nurse typhoid cases to use this medicine, as constant exposure to the fever, but for a very few days, may bring on an attack. That typhoid fever is contagious to a certain extent, I presume, none will doubt. —*Southern Medical and Surgical Journal.*

